# A model of metformin mitochondrial metabolism in metachromatic leukodystrophy: first description of human Schwann cells transfected with CRISPR-Cas9

**DOI:** 10.1098/rsob.210371

**Published:** 2022-07-06

**Authors:** Nayibe Tatiana Sanchez-Álvarez, Paula Katherine Bautista-Niño, Juanita Trejos-Suárez, Norma Cecilia Serrano-Díaz

**Affiliations:** ^1^ Faculty of Medical and Health Sciences, Masira Institute for Biomedical Research, Universidad de Santander, Bucaramanga, Colombia; ^2^ Faculty of Health, Phd in Biomedical Sciences, Universidad del Valle, Cali, Colombia; ^3^ Research Center Floridablanca, Colombian Cardiovascular Foundation, FL, Colombia

**Keywords:** metachromatic leukodystrophy (MLD), metabolic activity, metformin, neurological lysosomal storage disease, sulfatide therapy

## Abstract

Metachromatic leukodystrophy is a neurological lysosomal deposit disease that affects public health despite its low incidence in the population. Currently, few reports are available on pathophysiological events related to enzyme deficiencies and subsequent sulfatide accumulation. This research aims to examine the use of metformin as an alternative treatment to counteract these effects. This was evaluated in human Schwann cells (HSCs) transfected or non-transfected with CRISPR-Cas9, and later treated with sulfatides and metformin. This resulted in transfected HSCs showing a significant increase in cell reactive oxygen species (ROS) production when exposed to 100 µM sulfatides (*p* = 0.0007), compared to non-transfected HSCs. Sulfatides at concentrations of 10 to 100 µM affected mitochondrial bioenergetics in transfected HSCs. Moreover, these analyses showed that transfected cells showed a decrease in basal and maximal respiration rates after exposure to 100 µM sulfatide. However, maximal and normal mitochondrial respiratory capacity decreased in cells treated with both sulfatide and metformin. This study has provided valuable insights into bioenergetic and mitochondrial effects of sulfatides in HSCs for the first time. Treatment with metformin (500 µM) restored the metabolic activity of these cells and decreased ROS production.

## Graphical abstract

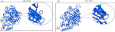

## 1. Introduction

Metachromatic leukodystrophy (MLD) is a lysosomal storage disease classified as an inborn error of metabolism that is inherited in an autosomal recessive manner. It is characterized by progressive demyelination of the central nervous system (CNS) and peripheral nervous system (PNS), causing severe neurological symptoms [1–3]. MLD is caused by different mutations in the arylsulfatase A gene, hereinafter referred to as *ARSA*, located on chromosome 22q13.33, comprising eight exons and encoding a 509 amino acid precursor protein [4,5].

To date, nearly 200 genetic mutations have been identified [6]. Most of them are nonsense mutations, thus leading to deficient expression or structural damage of *ARSA* [7]. ARSA is a lysosomal acid hydrolase that catalyses the first step in the degradation of cerebroside 3-sulfate, a sulfatide mainly found in CNS white matter and PNS [8,9].

The overall incidence of MLD remains unknown. Nevertheless, the Orphanet database reports an estimated prevalence of approximately one case per 625 000 live births and an incidence of 0.5 to 1 per 50 000 [1]. However, there is a lack of official records of people who have developed the disease or are at risk of developing it or transmitting it to their offspring [10].

Currently, the use of drugs whose mechanism of action is known to counteract other diseases is in place. This is the case of metformin, which is the main first-line oral hypoglycaemic drug of choice in patients with type 2 diabetes worldwide, and is also currently used for neurodegenerative diseases and cancer due to its ability to delay ageing in humans [11]. Although its mechanism of action is well known, its effects on cells remain unknown [12,13].

Cheng *et al*. [12] demonstrated that metformin acts through the v-ATPase-Ragulator lysosomal pathway to coordinate mTORC1 and AMPK [12]. Therefore, the main objective was to determine the effect of the use of metformin on the accumulation of sulfatide in glycolysis and mitochondrial function through an *in vitro* model of MLD.

Individuals diagnosed with MLD must face the ageing process every day, as they can develop symptoms, psychiatric disorders, and loss of motor, cognitive and social skills [14]. To date, there is no treatment or timely diagnostic scheme to control the evolution of symptoms and prevent the death of patients. The difficulty in developing an effective drug to treat MLD is mainly due to poor understanding of disease development, insufficient reporting of molecular mechanisms and lack of interest in rare diseases by the pharmaceutical industry [2]. This research has an impact on the analysis of new empiric treatments in MLD and how the Schwann cell mitochondrial metabolism responds to metformin use.

Considering the behaviour of sulfatide in other systems, with the development of this project it was possible to demonstrate that sulfatide accumulation alters cellular metabolism, such as glycolysis and mitochondrial function, in myelinating PNS cells, as in the case of ARSA-deficient cells. We evaluated the capacity of metformin to reverse the effects of sulfatide accumulation.

## 2. Material and methods

### 2.1. Cell culture

Human Schwann cells (HSCs) isolated from human spinal nerve (ScienCell Research Laboratories [15]) were selected for this study. Cells were thawed to assess their viability using 0.4% trypan blue dye in a Neubauer chamber and then cultured in Schwann cell medium (MCS, ScienCell Research Laboratories) in an environment with 5% CO_2_ concentration under manufacturer's recommendations [16].

In addition, the experiments in this research were performed in triplicate for non-transfected cells and in duplicate for transfected cells, and repeated two and three times, respectively, to collect required data for conducting a more accurate analysis to answer the research questions related to the disorder described herein and the method implemented to improve this condition.

### 2.2. Cell transfection

Cultured HSC density increased between 30% and 70% 24 h after seeding on assays, which corresponds to approximately 2 × 10^4^ cells per well on 24-well plates (Corning, Life Sciences). The CRISPR-Cas9 assay was performed with 500 µl of MCS. In addition, ribonucleoprotein complex assembly was performed at a ratio of 1.3 : 1 of custom single guide RNA containing a targeting sequence (sgRNA). GeneArt Platinum Cas9 nuclease (Life Technologies) and Lipofectamine CRISPRMAX Cas9 Transfection Reagent (Invitrogen) were used according to the manufacturer's instructions. sgRNA (GACCGUGGCCGAAGU) (Synthego) is a 20-nucleotide gene sequence that is homologous to exon 2 of the ARSA gene directing Cas9 nuclease activity. sgRNA sequence was used to align two or more sequences (https://blast.ncbi.nlm.nih.gov/Blast.cgi) [17] to *Homo sapiens* chromosome 22 (NC_000022.11) [2] for analysing and positioning guide RNA sequences and/or primers.

Cells cultured in the 24-well plate were trypsinized and 50 µl of the sgRNA and Opti-MEM mix in reduced serum medium, Lipofectamine Cas9 Plus Reagent, GeneArt Platinum Cas9 nuclease and Lipofectamine CRISPRMAX were added to the wells. Additionally, Lipofectamine transfection reagent was added to the transfection reagent according to the manufacturer's instructions [18]. Cells were incubated at 37°C in an atmosphere of 5% CO_2_ for 3 days. After incubation, clonal cell expansion was carried out by limiting dilution, incubated at 37°C in an atmosphere of 5% CO_2_ until 80% confluence and transferred to a 60 mm^3^ Petri dish for subsequent assays.

### 2.3. Real-time reverse transcriptase-polymerase chain reaction

ARSA expression in Schwann cells was quantified before and after transfection with CRISPR-Cas9. In addition, RNA extraction was performed using TRIzol reagent (Ambion). RNA concentration and quality were measured spectrophotometrically using a NanoDrop 2000 spectrophotometer (Thermo Fisher Scientific) at a A260/A280 ratio of 1.75. In addition, real-time reverse transcriptase-polymerase chain reaction (RT-qPCR) assays were performed using 2X Luna Universal Probe One-Step RT- qPCR (New England Biolabs), 0.4 µM predesigned forward/reverse TaqMan gene expression assay primers (Life Technologies) + FAM-labelled TaqMan probe fluorophore, 20X Luna WarmStart RT enzyme mix and sample RNA with the concentration of 100 ng µl^−1^ in a final volume of 20 µl. Thermal cycling was performed at 55°C for 20 min (reverse transcription), followed by 45 cycles of 95°C for 5 min, 95°C for 15 s, 60°C for 45 s and a final extension at 60°C for 5 min for a 69 bp amplicon. Results were analysed using Bio-Rad CFX Manager version 3.1.1517.0823 [19].

To determine the number of copies expressed in the *ARSA* gene of transfected cells compared to non-transfected cells, the relative quantification method was analysed, using endogenous control genes glyceraldehyde-3-phosphate dehydrogenase with the formula:

$$\begin{array}{rl} & \Delta Ct\;Transfected\;cells=\Delta Ct\;ARSA\;gene-\Delta Ct\;GAPDH\;gene\\ & \Delta Ct\;Untransfected\;cells\;ct=\Delta Ct\;ARSA\;gene-\Delta Ct\;GAPDH\;gene\\ & \Delta GDCt\;Transfected\;cells=Untransfectd\;cells\;\Delta \Delta cc \end{array}$$

### 2.4. Sequencing and bioinformatics analysis

Before sequencing exon 2 of the *ARSA* gene for the analysis of mutation in transfected cells, conventional PCR amplification was performed in a final volume of 50 µl with 2X Master Mix Go Taq Green (Promega), 10 nM of primer forward 5′CCTACCTGGTCGGAGTA3′, primer reverse 5′TGTCCCGCAGGCAGGCCG3′ and 100 ng of DNA. Thermal cycling was performed at 94°C for 5 min, followed by 35 cycles of 94°C for 30 s, 60.5°C for 40 s, 72°C for 30 s and 72°C for 5 min. Electrophoresis was performed on a 1.0% agarose gel at 80 volts for 45 min to obtain a 256-base pair (bp) band. PCR products were sequenced by capillary electrophoresis (Macrogen Inc.). Once the sequence was obtained, the assembly was performed using SeqMan Ultra-LaserGene version 17 (DNASTAR) [20], which is homologous with exon 2 of the *ARSA* gene (NG_009260.2) [21]. Based on observed differences, a high-resolution three-dimensional theoretical structural model of the protein was developed using the Swiss Model Server Structure Evaluation Tool [22] by using the human arylsulfatase protein sequence as template A (PDB ID: 1AUK) [23].

### 2.5. Cell viability and cytotoxicity

Cell viability and proliferation were assessed by the 3-(4,5-dimethylthiazol-2-yl)-2,5-diphenyltetrazo (MTT) bromide method (Alpha Aesar). Cell cytotoxicity enabled determination of sulfatide and metformin concentrations for subsequent testing. In each assay, MCS was served as an untreated control to normalize treatment results.

HSCs were seeded in 96-well plates at a density of 2 × 10^4^ cells per well tested and incubated at 37°C in a 5% CO_2_ atmosphere for 24 h. The cells were exposed to 10, 25, 50 and 100 µM sulfatide (Matreya) in the same plate and exposed to 10, 25, 50, 100, 300 and 1000 µM metformin (1,1 dimethylbigunide hydrochloride, 97%, Acros Organics). After 24 h incubation, the medium was replaced with MTT dissolved in MCS at a concentration of 1 mg ml^−1^ for 1 h in a 5% CO_2_ atmosphere at 37°C, after which MTT was removed from each well and replaced with 100 µl of ACS grade dimethyl sulfoxide (Amresco). Colour intensity was measured spectrophotometrically by a Varioskan Flash microplate reader Thermo Fisher Scientific) at a wavelength of 570 nm.

For analyses, mean, standard deviation and level of cytotoxicity were determined in accordance with ISO 10993-5:2009, following the classification of cytotoxicity scores according to the percentage of viable cells: 71–100% for non-cytotoxic and less than 70% for potentially cytotoxic [24].

### 2.6. Evaluation of cell death

Non-transfected and transfected cells were cultured in 96-well plates at a density of 3 × 10^4^ cells per well at 37°C for 2 h in a 5% CO_2_ atmosphere for subsequent treatment with different concentrations of sulfatide and metformin dissolved in the culture medium. After treatment, they were incubated at 37°C in a 5% CO_2_ atmosphere for 24 h. On the day of the assay, cell death was determined by incubating cells with 0.05 µM SYTOX Green nucleic acid stain (Invitrogen) dissolved in MCS (MCS-SYTOX). One hundred microlitres of MCS-SYTOX was added to each well, followed by an incubation for 15 min during which the Varioskan Flash microplate reader (Thermo Fisher Scientific) was used to determine fluorescence.

To determine the number of dead cells and average fluorescence of wells in triplicate, the ratio between control well fluorescence and fluorescence of wells containing lysed cells in the presence of 0.1% Triton X-100 (Amresco) was used as a positive and negative control with MCS. Increased fluorescence is associated with increased cell death.

### 2.7. Apoptosis

Apoptosis was assessed using caspases 3/7 and annexin V assays to differentiate apoptotic and necrotic processes. HSCs were seeded until reaching 70% confluence. Caspase-3 and -7 activities were determined using the CellEvent Caspase- 3/7 Green Flow Cytometry Assay Kit (Life Technologies).

In addition, HSCs were treated with 100 µM sulfatide and 500 µM metformin in culture and incubated for 24 h at 37°C with a CO_2_ concentration of 5%. Four micromoles of doxorubicin was used as a positive control (Ebewe). Cytometry tubes each containing 1 ml of cell suspension in phosphate-buffered saline were treated according to the manufacturer's instructions [25]. Finally, samples were analysed using a 488 nm excitation filter, 530/30 (green) emission filter for CellEvent reagent and 690/50 (red) filter for SYTOX AADvanced. Cell viability was measured with a FACScan III flow cytometer (BD Biosciences). A minimum of 2000 events were recorded.

Phosphatidylserine translocation from the inner to the outer leaflet of the cellular membrane as a differentiator of apoptosis and mitochondrial membrane potential in live cells was evaluated using the MitoTracker Red and Alexa Fluor 488 Annexin V kit (Invitrogen) by flow cytometry using the FACScan III kit (BD Biosciences) to acquire 2000 events according to the manufacturer's instructions [26]. This assay is based on the detection of phosphatidylserine translocation and changes in mitochondrial membrane potential.

Apoptosis was induced in HSCs after treatment with 10, 25, 50 and 100 µM sulfatide, 500 µM metformin, 100 µM sulfatide and 500 µM metformin. Negative control results were prepared by incubating the cells in the absence of any inducing agent. These cells were incubated using 4 µM doxorubicin as a positive control for necrosis (Ebewe).

In addition, apoptotic cells show a strong green fluorescence with decreased red fluorescence in comparison to very little green fluorescence and bright red fluorescence in living cells. These populations can be easily distinguished using a flow cytometer to measure fluorescence emission spectra at 530 and 585 nm.

### 2.8. Mitochondrial bioenergetics

As recommended in the Mito Stress Test Kit protocol (Agilent Technologies) [27], the standardization of the number of cells and the concentration of carbonylcyanuro-p-trifluoromethoxyphenylhydrazone (FCCP, ChemScene) were carried out in response to this uncoupler to ensure optimal and reproducible culture conditions for comparability between data and scientific results collected from HSC. Therefore, the total cell population was 50 000 cells.

Non-transfected and transfected HSCs were cultured in Seahorse 24-well plates (Agilent Technologies) at a density of 5 × 10^4^ cells in 100 µl of MCS and treated with 150 µl of 10, 25, 50 and 100 µM sulfatide and 500 µM metformin for 24 h at 37°C with 5% CO_2_ for 2 h. The mitochondrial bioenergetic function was determined using the XF Cell Mito Stress Test kit to measure mitochondrial metabolism (Agilent Technologies) using extracellular flux analysis on a Seahorse XFe24 Analyzer (Agilent Technologies) following the manufacturer's instructions [27].

At the end of each assay run, the Bradford assay was used to determine protein concentration. To normalize the assay, proteins were read at a wavelength of 590 nm using a Thermo Scientific Varioskan Flash plate reader (Thermo Fisher Scientific).

One hour before starting the experiment, HSCs were washed and replaced with DMEM (Caisson Labs) without buffer, supplemented with 1 mM pyruvate, 10 mM glutamine and 5.5 mM glucose, and the medium was adjusted to pH 7.4. After establishing the baseline cellular oxygen consumption rate and extracellular acidification rate (ECAR), metabolic changes are measured by adding inhibitors, 1.5 µM oligomycin (ChemScene) and 1 µM FCCP and antimycin A/rotenone (ChemScene).

### 2.9. Mitochondrial reactive oxygen species

Mitochondrial reactive oxygen species (ROS) levels were measured using a MitoSOX Red fluorescent probe (Invitrogen) following the manufacturer's instructions [28]. Fluorescence intensity was measured using an Eclipse Ti-S inverted microscope (Nikon) at a 510/580 nm for emission/excitation.

### 2.10. Statistical analysis

Statistical analyses were performed using GraphPad Prism 8 (GraphPad Software) [29]. All data are presented as the mean ± s.e.m. Statistical differences between both groups were analysed using *t*-tests for unpaired data. When evaluating both groups, these were analysed by one-way analysis of variance using the Bonferroni test. *p*-values of *p < 0.05, **p < 0.01 and ***p < 0.001 were considered statistically significant.

## 3. Results

### 3.1. Cell transfection

In the analysis of results, cell transfection, RT-qPCR, and bioinformatics analysis and sequencing were discussed jointly, showing the characteristics of these analyses in both transfected and non-transfected cells.

First, cell transfection shows that transfected cells were obtained using the CRISPR-Cas9 system, modified for the *ARSA* gene, and all tests were based on the original *ARSA* gene sequencing (Gene ID: 410) [30] to ensure that both cell populations were functional (transfected and non-transfected cells).

Such genetic modification was confirmed by the gene variant reported in the Leiden Open Variation Database (LOVD3 whole-genome sequencing dataset) [24,31], which is homologous to the variant defined and recorded on 16 February 2016, with a single-nucleotide polymorphism (SNPrs745 884 435 recorded at position chr22: 50 627 213–50 627 219 [32]. In this case, the allele was a deletion of the guanine base (delG)-NM_000 487.6, causing a frameshift mutation [31] that is probably and pathologically associated with infantile MLD. The generation of mutations in Schwann cells allowed *in vitro* experimental simulation of the dynamic of Schwann cells in patients with MLD.

Second, real-time reverse transcriptase polymerase chain reaction (RT-qPCR) allowed us to verify the expression of the ARSA gene, whose result was 42.78 ± 0.07 s.d. in transfected cells versus 33.36 ± 0.03 s.d. in non-transfected cells with ARSA expression, which decreased by 183.5 copy numbers in transfected cells compared to non-transfected cells.

Finally, sequencing and bioinformatics analysis where the presence of ARSA was verified in both transfected and non-transfected cells using conventional PCR amplification and sequencing analysis showed 99% identity with the sequence of exon 2 of the ARSA gene (ID NG_009260.2), in which c.418del, a mutation previously reported as NM_000487. 6: c.418del [33], was identified.

**Figure 1.**
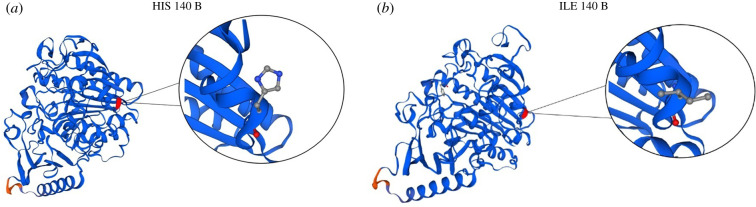
*In silico* protein modelling. Theoretical three-dimensional ARSA protein structure in (*a*) non-transfected Schwann cells (H140) and (*b*) transfected Schwann cells (I140).

Theoretically, this deletion causes a change in the protein (p.His140fs; dbSNP:rs745884435) [19]: H140 [CAT] > I140 [AT]. In molecular modelling of the three-dimensional structure of the protein used to compare structural similarities, a difference in protein folding was evidenced whenever an amino acid change occurred at this position (figure 1).

### 3.2. Cell viability and cytotoxicity

Cell viability was 100% in non-transfected Schwann cells exposed to different concentrations of 10–100 µM sulfatide and 10–1000 µM metformin for 24 h.

It should be noted that, to evaluate the effect of sulfatide accumulation in transfected and non-transfected Schwann cells, it is essential to determine cell cytotoxicity using an MTT assay, as well as sulfatide and metformin treatments used in other assays proposed for this research. According to the results obtained from the exposure of both cells populations to sulfatides and metformin, it was possible to select concentrations that did not produce cell death.

**Figure 2.**
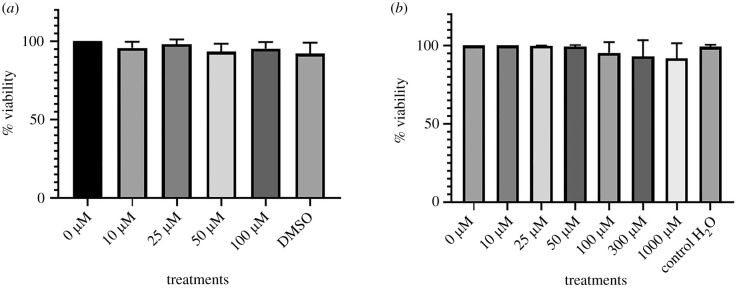
MTT test. (*a*) Non-transfected Schwann cells were exposed to 10, 25, 50 and 100 µM sulfatide concentrations for 24 h in 5% CO_2_ atmosphere. (*b*) Transfected Schwann cells were exposed to 10, 25, 50, 100, 100, 300 and 1000 µM metformin for 24 h in a 5% CO_2_ atmosphere.

Figure 2 shows the percentage of cell viability in non-transfected Schwann cells exposed to concentrations of 10 to 100 of sulfatides and 10 to 100 µM of metformin for 24 h. In this assay, the treatment using selected sulfatides and metformin concentrations are observed to not cause cell death.

### 3.3. Evaluation of cell death

**Figure 3.**
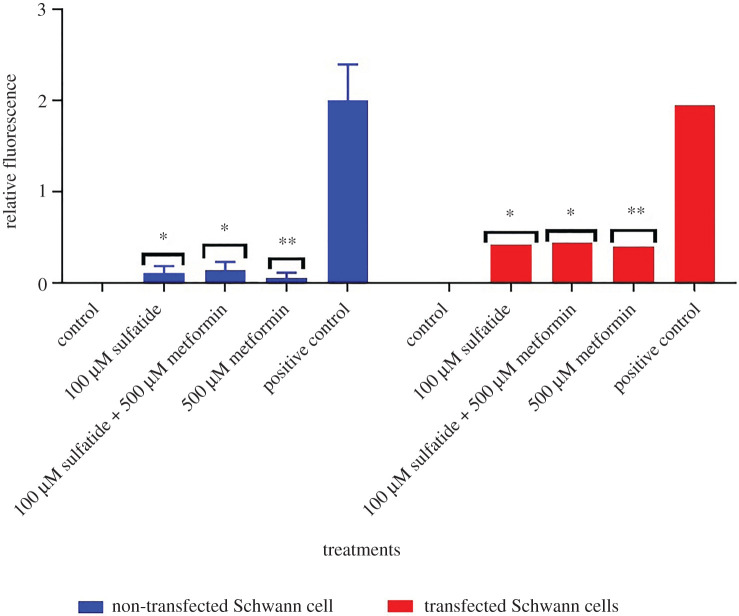
Cell death was observed in transfected and non-transfected Schwann cells. Relative fluorescence of transfected and non-transfected Schwann cells at different concentrations of sulfatides stained with SYTOX Green.

**Figure 4.**
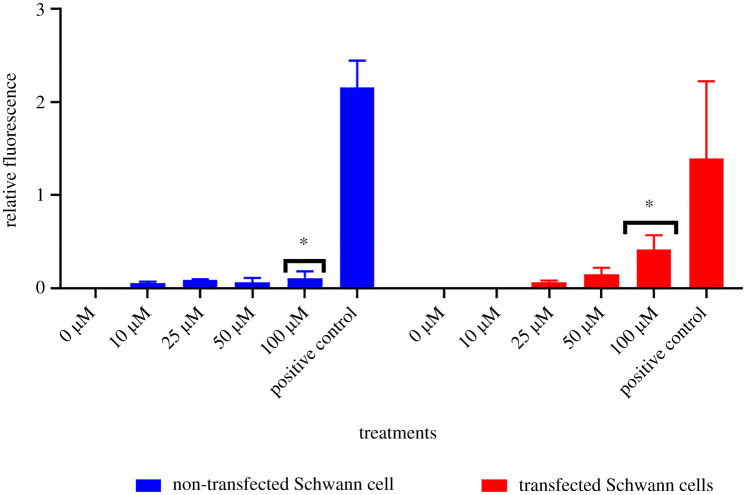
Cell death was observed in transfected and non-transfected Schwann cells. Relative fluorescence of transfected and non-transfected Schwann cells at different concentrations of SYTOX Green Stain. Cells lysed in the presence of 0.1% Triton X-100 are assayed as a positive control and cells from culture medium as a negative control. Sulfatide concentrations were not observed as no statistically significant differences were found.

As transfected and non-transfected Schwann cells were treated using concentrations of 10, 25, 50 and 100 µM of sulfatides, cell death increased, although a statistically significant difference of *p* = 0.0124 was found exclusively in the 100 µM concentration between transfected and non-transfected Schwann cells (figure 3). Therefore, this concentration was chosen to identify whether metformin could mitigate cell death by sulfatide accumulation in transfected Schwann cells. In addition, statistically significant differences of *p* = 0.0124 were found in transfected cells over the three treatments when using 100 µM of sulfatides (*p* = 0.0241), combined treatment with sulfatide and metformin and 500 µM of metformin (*p* = 0.0042), (figure 4).

### 3.4. Apoptosis

One hundred micromoles of sulfatide did not activate caspase 3/7 in non-transfected Schwann cells, which is used as a measure of apoptotic or necrotic cell death. Similarly, 100 µM of sulfatide induced an increase in necrotic cell death in transfected Schwann cells. In addition, simultaneous treatment with sulfatide and metformin enhanced apoptosis and necrosis in these cells.

**Figure 5.**
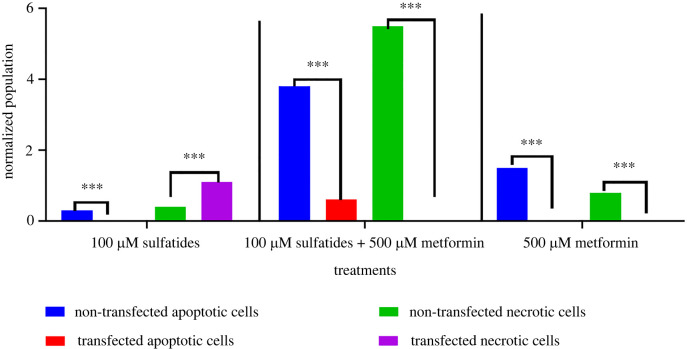
Cell apoptosis by flow cytometry. Data are representative of two studies conducted in independent assays. ***p < 0.001.

The analysis of cell apoptosis by flow cytometry showed that both transfected and non-transfected Schwann cells were exposed to 100 µM sulfatide or 100 µM sulfatide + 500 µM metformin but only 500 µM metformin showed a statistically significant effect difference between apoptotic and live cells (figure 5).

### 3.5. Mitochondrial bioenergetics

**Figure 6.**
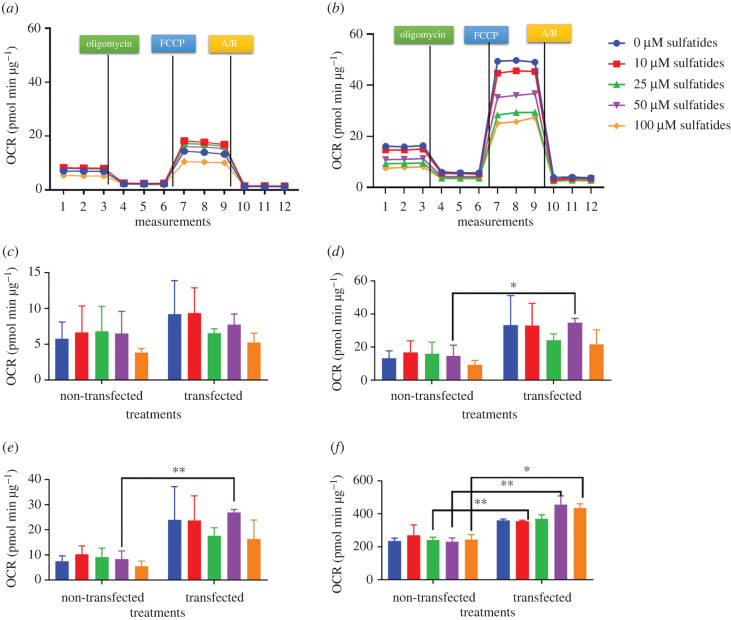
Mito stress test, OCR in transfected and non-transfected Schwann cells. Data are representative of two independently conducted assays. *p < 0.05, **p < 0.01, ***p < 0.001. Maximal respiration, *p* = 0.0277; spare respiratory capacity, *p* = 0.0053; spare respiratory capacity % 25 µM, *p* = 0.0053; 50 µM, *p* = 0.0061; 100 µM, *p* = 0.0210. (*a*) OCR non-transfected Schwann cell, (*b*) OCR transfected Schwann cell, (*c*) basal respiration, (*d*) maximal respiration, (*e*) spare respiratory capacity and (*f*) spare respiratory capacity %.

The ideal FCCP concentration was 0.5 µM so that maximal oxygen consumption and basal respiration would not be less than 20 pmol min µg^−1^. Using the XF Cell Mito Stress Test Kit, non-transfected Schwann cells exposed to sulfatides at concentrations of 10, 25 and 50 µM were able to show metabolic response as evidenced by increased maximal respiration. However, this response did not occur when cells were exposed at a concentration of 100 µM as their basal oxygen consumption, maximal respiration and reserve capacity decreased (figure 6).

When comparing the results with those of transfected Schwann cells, these cells showed an increase in maximal respiration without sulfatide exposure. However, as sulfatide concentration increased, respiration and basal respiration were affected (figure 6).

**Figure 7.**
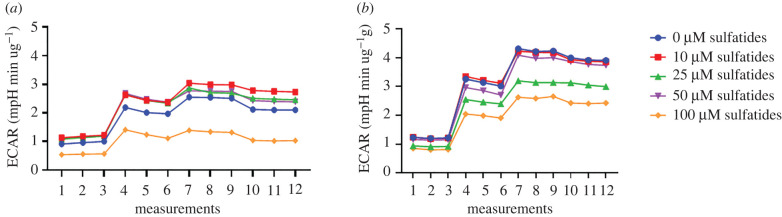
Mito stress test, ECAR rates of transfected and non-transfected cells. (*a*) Non-transfected Schwann cells. (*b*) Transfected Schwann cells.

Similarly, transfected Schwann cells showed an increase in ECAR rates compared to non-transfected Schwann cells (figure 7).

**Figure 8.**
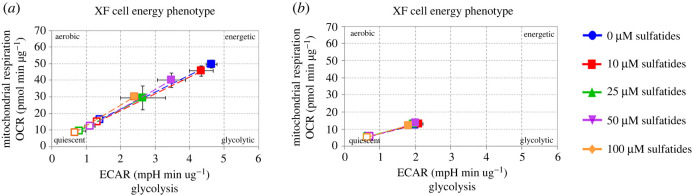
Energy phenotype in transfected and non-transfected Schwann cells treated with sulfatides. (*a*) Non-transfected Schwann cells. (*b*) Transfected Schwann cells.

Differences were observed in energy phenotype maps between the two groups of cells (figure 8*a,b*) and an increase in the glycolytic pathway activity, which is in response to mitochondrial stressors, as observed in the statistical analysis of the energy phenotype (figure 8). Increased ECAR levels were reported as 2.10, 1.95, 2.01 and 1.81 mpH min ug^−1^ with respect to 10, 25, 50 and 100 µM sulfatide treatment for non-transfected cells, respectively, and 4.34, 3.47, 3.47 and 2.41 mpH min ug^−1^ for transfected cells, respectively.

### 3.6. Metformin in sulfatide metabolism

**Figure 9.**
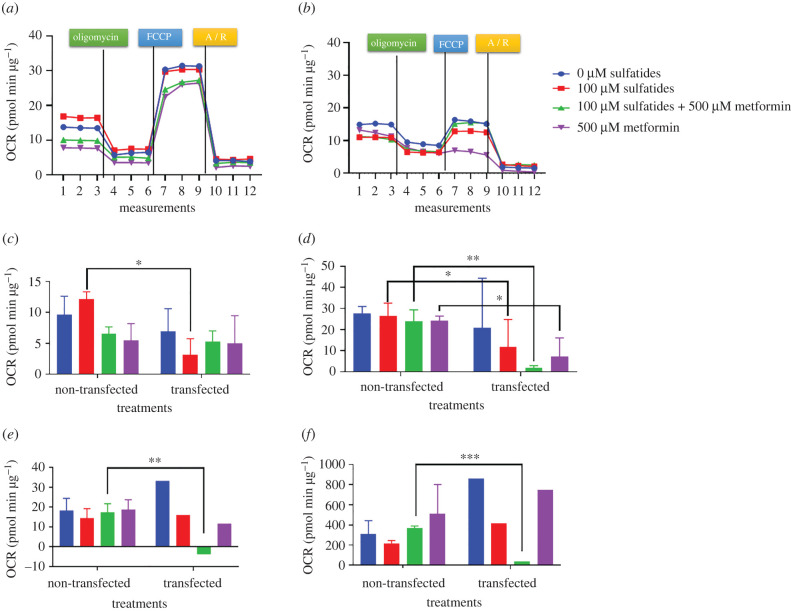
Mitochondrial bioenergetics with metformin pulldown. XF Cell Mito Stress Test Kit Assay in (*a*) non-transfected cells and (*b*) transfected cells. Parameters of mitochondrial respiration, basal respiration (*c*) maximal respiration (*d*) maximal respiration, (*e*) normal reserve respiratory capacity (*f*) and in per cent. Data are representative of two independently performed assays. *p < 0.05, **p < 0.01, ***p < 0.001. Basal respiratory, *p* = 0.0470; maximal respiration, 100 µM, *p* = 0.0285; 100 µM sulfatide + 500 µM Metformin, *p* = 0.0048; 500 µM Metformin, *p* = 0.0406.

Non-transfected cells were exposed to 100 µM sulfatide, in which metformin treatment did not affect mitochondrial respiration (figure 9*a*). By contrast, as transfected cells did not respond to sulfatide exposure, a decrease in both basal respiration and maximal mitochondrial respiration, as well as mitochondrial respiratory capacity and percentage, was observed (figure 9*c*–*f*). In addition, simultaneous treatment with 100 µM sulforaphane and 500 µM metformin improved mitochondrial energy production in transfected cells (figure 9*b*). The above indicates that mitochondrial function was impaired at the expense of sulfatide accumulation. However, ATP was constantly produced in both groups of cells.

**Figure 10.**
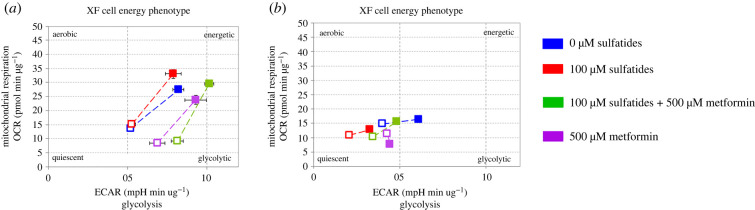
Phenotype map of metformin treatment. (*a*) Non-transfected Schwann cells. (*b*) Transfected Schwann cells.

Phenotypic profiles of metformin in transfected and non-transfected cells were evaluated. Non-transfected cells showed to have an energy phenotype (figure 10*a*), while transfected cells that tended to be quiescent when exposed to 100 µM sulfatide and cells treated simultaneously with sulfatide and metformin recovered their initial energy profile. Cells treated with metformin only are considerably more glycolytic (figure 10*b*). Transfected cells exposed to 100 µM sulfatide showed more metabolic alterations.

### 3.7. Mitochondrial reactive oxygen species

**Figure 11.**
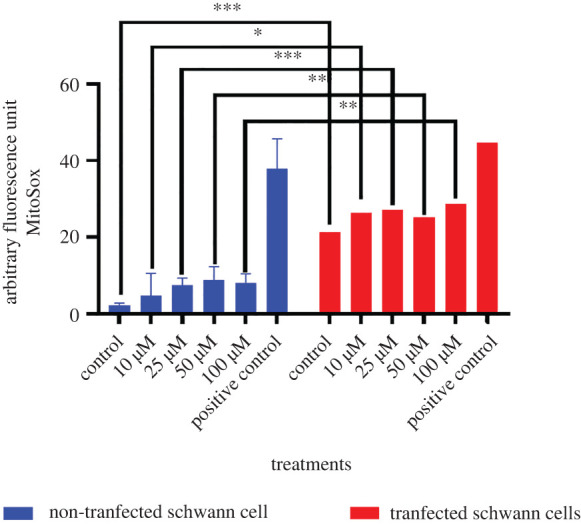
Quantification of mitochondrial superoxide anion in Schwann cells. Statistical significance was calculated by applying the Student's test. *p < 0.05, **p < 0.01, ***p < 0.001 for each of transfected and non-transfected cells. Control, *p* < 0.0001; 10 µM, *p* *=* 0.0159; 25 µM, *p* *=* 0.0008; 50 µM, *p* *=* 0.0083; 100 µM, *p* *=* 0.0012.

Statistically significant differences were observed between the two groups of cells (figure 11). Transfected cells exposed to 100 µM sulfatide produced superoxide anions.

**Figure 12.**
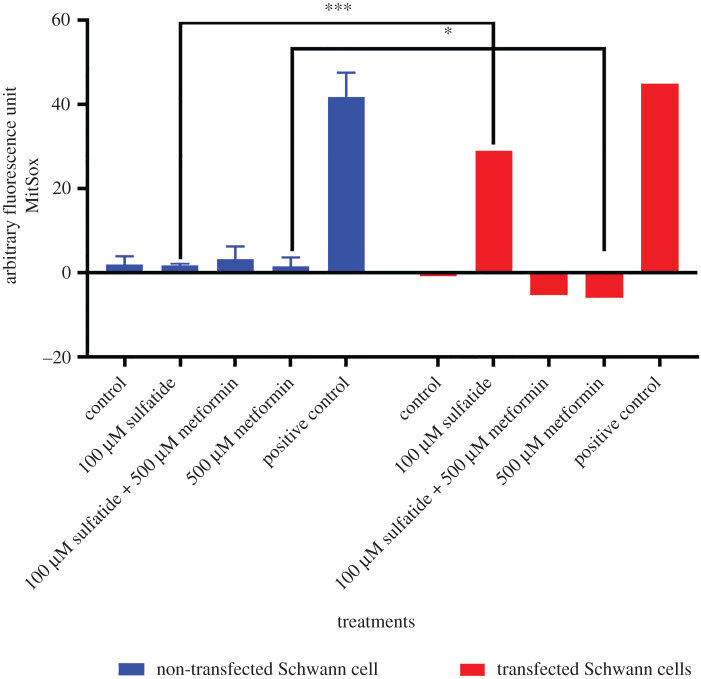
Decrease in mitochondrial superoxide production. Statistical significance was calculated by applying the Student's test. *p* = 0.0414 (*p < 0.05), *p* = 0.0001 (***p < 0.001) for each of the concentrations compared between transfected and non-transfected cells.

Treatment with metformin (500 µM) prevented the formation of superoxide anions in transfected cells (figure 12).

**Figure 13.**
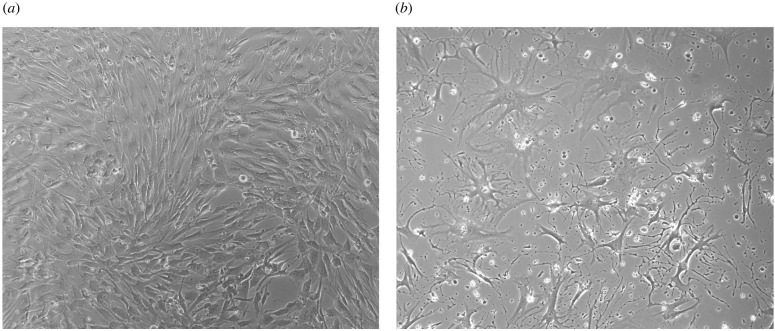
(*a*) Phenotypic characteristics of non-transfected cells; (*b*) Phenotypic characteristics of transfected cells.

All these experiments were performed in triplicate and repeated in three independent assays for non-transfected cells. For transfected cells, due to the CRISPR-Cas9 gene editing that decreases the viability of these cells, only one assay was performed (see figure 13*a*,*b*).

## 4. Discussion

MLD is a lysosomal storage disease leading to sulfatide accumulation due to a deficiency of the lysosomal enzyme ARSA, causing clinical manifestations characterized by progressive motor and cognitive deficits. The severity of the clinical course of MLD is determined by the residual ARSA activity, depending on the type of mutation [34]. Despite the efforts made to identify effective treatments for MLD, there are currently no effective therapeutic options available. Existing treatments, such as bone marrow or umbilical cord blood transplantation, cannot prevent disease progression [6]. Therefore, complementary therapies are required to improve the quality of life in patients with MLD. In this study, a genetic modification was performed by CRISPR-Cas9 genome editing using a lipid-based transfection procedure, in addition to a mutation in exon 2 of the ARSA gene in HSCs (ScienceCell Research Laboratories, USA), which is a method that has not been described in this cell type so far.

In addition, analysis of the theoretical three-dimensional structure model showed that the deletion of guanine nucleotide causes a premature termination codon within a protein in which the amino acid histidine at position 140 changes to isoleucine (H140I) [7]. The mutation found in this study is correlated with the heterozygous variant used as a positive control in the identification of infantile MLD according to the cohort study of McCreary *et al*. [35], in which a targeted approach using a panel of 257 genes was developed for a population of 60 children with suspected genetic neuroinflammation, which allowed the confirmation of molecular diagnosis in 20% of patients, and which outlined some unexpected genotype-phenotype associations and new pathogenic variants.

In our model of transfected Schwann cells with no ARSA activity, gene expression was analysed using RT-qPCR, showing a lower expression compared to the copy number expressed in non-transfected cells, which indicates that amino acid changes significantly affected the structure and function of ARSA protein. The above is in accordance with a study conducted by Guo *et al*. that described *ARSA* overexpression in mutant or transfected cells showing enhanced efficacy of ARSA enzyme against sulfatide metabolism [36–39].

The enzyme ARSA is responsible for the metabolism of the sphingolipid 3-o-sulfogalactosylceramide, known as sulfatide, which prevents its accumulation in lysosomes. This lipid plays an important role in both CNS and PNS in the myelination process. Therefore, a mutation in the gene encoding this enzyme would lead to an imbalance in this process [40]. Selected concentrations in this study that do not affect cell viability in Schwann cells were 10, 25, 50 and 100 µM based on the studies conducted by Blomqvist *et al*. [41], in which the developmental profile of lysosulfatide in the brain of ARSA-deficient mice was evaluated, in addition to the studies of Dali *et al.* [42] in which the accumulation of sulfatides and lysosulfatides in nerves and cerebrospinal fluid provides a marker of disease severity in the PNS only. C16:0 sulfatide (Matreya, Pleasant Gap, PA) in the range of 20–2000 ng mg^−1^ of dry tissue and 400 ng mg^−1^ of C12 : 0 sulfatide (ISTD, Avanti Polar Lipids) [42,43] were both used in this study.

Therefore, after selecting concentrations, the effects of different concentrations of sulfatides on transfected and non-transfected Schwann cells were evaluated. The viability of transfected Schwann cells was affected after exposure to 100 µM sulfatide. These data can be compared with the results obtained in other studies in which there was a correlation between the phenotype of ARSA-deficient mice observed and high levels of sulfatide [44]; Shaimardanova *et al*. [32], Rosenberg *et al*. [45] and Beerepoot *et al*. [46] confirmed that the presence of high concentrations of sulfatides in the CNS and PNS leads to demyelination due to the damage to myelin sheath covering most nerve fibres [34,45,46]. This damage is influenced by the accumulation of undigested lipids in the lysosome, which may invade other cell organelles, leading to enzyme deficiencies and triggering cell death [34].

Deficiencies of lysosomal enzymes lead to the development of lysosomal storage diseases, such as MLD, considering that these organelles are involved in a series of processes such as apoptosis and necrosis [47,48]. Necrosis was observed when Schwann cells were transfected after exposure to 100 µM sulfatide, while apoptosis was observed when cells were simultaneously exposed to 100 µM sulfatide and 500 µM metformin. The above is consistent with the fact that each type of cell in the human body has a different metabolism and therefore reacts according to the need for substrate. There is little information on MLD physiological processes [49] using sulfatide in pancreatic β-cells at 30 µM, which significantly reduces apoptosis, cell leakage and NO production [43].

Considering that the accumulation of metachromatic material in peripheral nerves in MLD has been previously reported, metachromatic material comprises Schwann cells and endoneurial macrophages that are filled with characteristic lysosomal sulfatide inclusions, also known as inclusion bodies [3,4]. The presence of sulfatide in these cells causes cell death and thus demyelination, leading to the onset of symptoms in patients with MLD [46].

This is the first study describing mitochondrial behaviour in live HSCs (non-transfected and transfected cells). Therefore, at the phenotypic level, transfected cells were quiescent when exposed to different concentrations of sulfatides, in contrast with non-transfected cells, as these showed an energetic profile found in the mitochondrial phenotype, in which transfected cells cannot use the metabolic pathways of glycolysis and oxidative phosphorylation to meet their energy demands under stress conditions. This also correlates with the results of ROS generation and necrosis due to the same mitochondrial involvement [50].

When evaluating the global profiling of ROS in transfected Schwann cells, we found that these cells produced higher levels of superoxide at the mitochondrial level compared to non-transfected cells as transfected cells were exposed to different concentrations of sulfatides, which was statistically significant. However, the use of metformin decreased this production.

The positive charge on the phosphonium group in MitoSOX Red selectively directs this cell permeable HE derivative to mitochondria, where it accumulates as a function of the mitochondrial membrane potential and shows fluorescence upon oxidation and subsequent binding to mtDNA [51].

The generation of ROS and reactive nitrogen species (RNS) is an integral process in cellular functions. ROS and RNS include various chemicals with different reactivity, such as superoxide anion radicals (O_2_^–^), hydrogen peroxide (H_2_O_2_), peroxynitrite (ONOO^–^), hydroxyl radicals (OH), nitrogen dioxide radicals (NO_2_) and carbonate anion radicals (CO_3_^–^). ROS and RNS have been proposed as these play an important role in the regulatory mechanisms, biochemical signal transduction and defence response against microorganisms. However, excessive production and/or insufficient detoxification can lead to oxidative/nitrative damage via ROS and RNS, which induces modification of cellular components, including proteins, lipids and DNA [52].

Oxidative stress has been described as an imbalance in the generation and neutralization of ROS and RNS in living organisms, leading to the overproduction of steady-state ROS and RNS. Oxidative stress alters redox homeostasis in several diseases, such as atherosclerosis, cancer, neurodegenerative diseases and myocardial infarction, which may cause irreversible damage and exacerbate a disease state [53,54]. Similarly, defective autophagy leads to the accumulation of mitochondria within which ROS can be generated due to the exposure to cellular stress [55].

The treatment for MLD remains enigmatic. However, efforts should continue to focus on identifying alternative treatments to improve the quality of life of patients. Here, metformin was proposed as a treatment to ameliorate and reduce the effects caused by sulfatide accumulation at the mitochondrial level.

Metformin is a plant-based drug that has been widely used to treat diabetes since the 1950s [56]. This drug was chosen because, although it is a biguanide used as first-line treatment of type 2 diabetes, it has been widely proposed as an alternative treatment for other pathologies, such as Parkinson's disease [57], Alzheimer's disease [58], liver disease [13] and multiple sclerosis [59], among others, because it has multiple antioxidant, anti-inflammatory, anti-apoptotic and anti-cancer properties [60–62]. Metformin has been found to cross the blood–brain barrier and accumulate in the brain *in vivo* [63].

Metformin has been shown to act via both AMP-activated protein kinase (AMPK)-dependent and AMPK-independent mechanisms, and by inhibition of mitochondrial respiration, as well as by inhibition of mitochondrial glycerophosphate dehydrogenase and a mechanism involving the lysosome [13,64]. Labuzek *et al*. [63] demonstrated that metformin alters lysosomal pH, thereby activating lysosomal enzymes in microglia [63].

Treatment of transfected cells with metformin resulted in an increase in maximal mitochondrial respiration rates, as shown by normalization of the Seahorse XF Mito Cell Mito Stress test, compared to the decrease in this parameter evidenced in cells transfected and treated with sulfatide, indicating that this drug enhanced the response of mitochondrial metabolism. Similarly, a reduction in intracellular and mitochondrial ROS generation was observed.

## 5. Conclusion

The generation of transfected HSCs has been described for the first time, and the presence of sulfatides metabolically affects these cells at the mitochondrial level. Treatment with 500 µM metformin reduced ROS generation in cells and at the mitochondrial level. Metformin improved mitochondrial bioenergetic performance in cells harbouring ARSA mutations.

## Data Availability

The datasets generated and/or analysed during this study are publicly available on the Open Science Framework (OSF) website [12].
